# The democratic fallacy in matters of clinical opinion: implications for analysing cause-of-death data

**DOI:** 10.1186/1742-7622-8-1

**Published:** 2011-01-11

**Authors:** Peter Byass

**Affiliations:** 1 Umeå Centre for Global Health Research, Department of Public Health and Clinical Medicine, Umeå University, Umeå 90185, Sweden

## Abstract

Arriving at a consensus between multiple clinical opinions concerning a particular case is a complex issue - and may give rise to manifestations of the democratic fallacy, whereby a majority opinion is misconstrued to represent some kind of "truth" and minority opinions are somehow "wrong". Procedures for handling multiple clinical opinions in epidemiological research are not well established, and care is needed to avoid logical errors. How to handle physicians' opinions on cause of death is one important domain of concern in this respect. Whether multiple opinions are a legal requirement, for example ahead of cremating a body, or used for supposedly greater rigour, for example in verbal autopsy interpretation, it is important to have a clear understanding of what unanimity or disagreement in findings might imply, and of how to aggregate case data accordingly.

In many settings where multiple physicians have interpreted verbal autopsy material, an over-riding goal of arriving at a single cause of death per case has been applied. In many instances this desire to constrain findings to a single cause per case has led to methodologically awkward devices such as "TB/AIDS" as a single cause. This has also usually meant that no sense of disagreements or uncertainties at the case level is taken forward into aggregated data analyses, and in many cases an "indeterminate" cause may be recorded which actually reflects a lack of agreement rather than a lack of data on possible cause(s).

In preparing verbal autopsy material for epidemiological analyses and public health interpretations, the possibility of multiple causes of death per case, and some sense of any disagreement or uncertainty encountered in interpretation at the case level, need to be captured and incorporated into overall findings, if evidence is not to be lost along the way. Similar considerations may apply in other epidemiological domains.

## Introduction

The concept of "a second opinion" is common in many professional spheres, including clinical practice. This may be a matter of seeking confirmation of an initial viewpoint, or looking for refutation of a doubted opinion. The interpretation of multiple opinions may not, however, be logically consistent, in that two coinciding opinions are often taken to represent "truth", while ignoring the possibility that both may be incorrect. Conversely, a minority opinion may actually represent "truth", even if not readily perceived as such. This is the basis of the democratic fallacy, which is manifested when a majority opinion is inferred to constitute some kind of truth. Stated in more general terms, if a panel of ten people are asked to express a preference between products A and B, and more than 5 express their preference for A, then the correct inference is that the majority of people prefer product A; but an incorrect inference would be that A is an inherently better product than B, and such an interpretation would be an example of the democratic fallacy.

In matters of clinical judgement, it is not uncommon to seek a second opinion (a process which might be instigated either by clinician or patient). However, in routine clinical practice, it is often unclear (at least in any formalised sense) as to how a conflict between different clinical opinions can or should be resolved [1,2]. In addition, some individual opinions may (often justifiably) be considered to be more authoritative or reliable, perhaps because of factors such as seniority or specialised experience. Hence, even where there are only two opinions, they may not count equally, and one may effectively "out-vote" the other, irrespective of "truth" in a particular case. This kind of pseudo-democracy can therefore lead to various manifestations of the democratic fallacy. When larger numbers of opinions are potentially available, such as in a ward round or case conference context, the potential for confusion between majority opinions and correct conclusions becomes greater [3].

This potentially dangerous combination of pseudo-democracy and clinical opinion sometimes intrudes into epidemiological research. Although quantitative health research is usually designed to be as objective and consistent as possible at all stages of the research process, it is often necessary to include the collection of clinical opinion at the individual case level as part of data gathering. A careful methodological approach for this key stage of the process is critically important, but not always carefully considered. If every case is assessed by the same clinician, the methodological issues are fairly simple, but the entire research comes to depend critically on the skill and consistency of that one observer. If each case is assessed by just one of several clinicians, then the democratic fallacy is not an issue, but there may be serious problems of inter- and intra-observer variation. However, what often happens (possibly in response to justifiable concerns about the consistency of single assessments) is that each case is assessed by multiple clinicians. Sometimes these may be quality-assured by insisting on independent, blind assessments; but then in cases where there are discordant opinions, rather vague processes of negotiating a common consensus or bringing in additional opinions (which may also be considered to carry more individual weight) to arrive at a final conclusion are often followed. The democratic fallacy can easily intrude into these processes.

These principles could apply to a range of epidemiological settings, such as clinical trials with entry or endpoint criteria defined in terms of clinical opinion, and even more generally to scientific peer-review. One specific domain in which the above issues are currently clouding research is in analysing cause-of-death data. In settings where physician certification of cause-of-death is commonplace or required, the opinion of a single physician is often taken to be sufficient, though in various jurisdictions there may be requirements for second opinions, for example before a body can be cremated [4]. Procedures regarding second opinions are often modified on the basis of notorious cases, for example that of Harold Shipman in England [5], and such medico-legal developments may have implications for the epidemiological analysis of cause-of-death data over time. However, of more concern in terms of non-standardised analytical approaches to cause-of-death data is the issue of how clinical opinion is used in the context of verbal autopsy (VA) techniques, as used in settings where deaths are not routinely physician-certified [6].

This article explores practices that have grown up around physician interpretation of VA data and argues for more consistent and logical approaches.

## Analysis

### Common practices in interpreting VA data

After someone dies in a setting where routine death certification is not carried out, a VA interview with family, friends or carers of the deceased can yield valuable information about the circumstances of death. There has been a lot of work put in to formulating and standardising VA interview instruments [7]. Much less consideration, however, has been given to standardising the interpretation of VA interview material into cause-of-death data. Although computer models can give highly standardised interpretations [8], many researchers still prefer the more subtle and nuanced approach that physician interpreters can bring to the process. But then the unresolved issues are how many physicians should interpret each case and how should conflicting opinions be resolved? A general principle seems to have grown up that using more than one physician interpreter per case is desirable, presumably to bring greater rigour to the process, although the justifications for doing so are usually not made explicit. Recently published examples of cause-specific mortality research relying on physician interpretation of VA material reveal a wide variation of approaches [9]. Joshi et al. have argued for using a single physician per case, but more on operational than scientific grounds [10], although they were working in a project that initially used two initial blinded assessors, who were over-ruled by a third assessor when they disagreed. Kahn et al. described initially using two blinded assessors, bringing in a third on disagreements, then a discussion among all three if two agreed; a cause of death was only recorded if overall consensus could be achieved [11]. Often the precise methods used to arrive at what sometimes appears to be the "holy grail" of a single, unambiguous cause for each case are not clearly defined [12].

Many of these approaches seem to have grown up out of unstated assumptions that VA approaches should try to mimic physician certification and coding, even if unable to do so with great accuracy. International standard procedures for death certification are set out within the ICD-10 system [13] but that system goes into levels of detail and diagnostic sophistication that are often not realistically achievable with VA data. ICD-10 expects the possibility of multiple causes of death per case, categorised according to complex schemas into a single underlying cause (which is envisaged as the cause to be used for epidemiological tabulations) and antecedent cause(s). There is no place in ICD-10 for carrying forward doubt as to particular individual causes on the part of certifying physicians into aggregated analyses of coded causes, being specifically prohibited: "Qualifying expressions indicating some doubt as to the accuracy of the diagnosis ... should be ignored" [13]. Similarly, it is not possible to have "cause A or cause B" as an underlying cause. WHO, in trying to establish international standards for VA [14] based on ICD-10, still makes the assumption that the outcome should be one ICD-10 underlying cause per VA case. Although in principle antecedent causes might also be derived from VA material, these principles seem to be boiled down in practice in many VA applications to training physician coders to arrive at a single cause of death per case. Consequentially, if more than one physician coder is used per case, potentially complex issues arise in terms of how to handle agreements and disagreements at the individual case level. Universally in the VA literature, multiple opinions that agree on the same cause of death for a particular case are interpreted as representing the "true" cause, irrespective of the possibility of the democratic fallacy manifesting in some cases, while procedures for handling discordant opinions vary as discussed above.

### Epidemiological analyses of cause-of-death data

A major objective of arriving at case-level cause(s) of death is to move on to epidemiological analyses of mortality patterns. Epidemiology specialises in the science of uncertainty, and so the clinically-perceived imperative, reflected in the ICD-10 "underlying cause" concept, for an unambiguous single cause of death per case is no longer entirely relevant. What is more important is to arrive at the most likely possible cause(s) and to understand something about the certainty with which these are relevant to particular cases, feeding all of this into aggregated results. Thus, if two physician assessors suggest different outcomes, it is probably more relevant in epidemiological terms to record both as possible causes, allocating a weighting of 50% to each, rather than trying to squeeze the different opinions into a single cause of death. This approach also eliminates the possible effect of the democratic fallacy, since there is no longer any requirement for consensus. Even if three assessors per case are used, with two agreeing and one dissenting, the same methodology can be applied with a 67%:33% weighting.

In some studies of VA material, cases not achieving consensus between assessors, for example instances where two initial assessors disagreed and a third assessor came up with a third cause of death, have been automatically designated as "undefined" or "indeterminate". This generally puts them into the same analytic category as cases where VA interviews were not carried out successfully or there were no witnesses to the circumstances of death [11]. This seems a very difficult methodology to defend scientifically, since several carefully considered case reviews, albeit with different conclusions, are surely not equivalent to missing data, and can easily be handled epidemiologically with partial weights assigned to different opinions. Even if one assessor rates a case as "indeterminate" and one or two others assign a particular cause, such a case can be handled as partly indeterminate and partly attributed to particular causes.

When VA material is interpreted by probabilistic computer models, the likelihood of putative cause(s) can be made more explicit and it is also possible to characterise quantitatively the overall lack of certainty for a case [8]. These kinds of output can be handled in a similar way as outlined above, with the additional possibility of characterising part of a case as "indeterminate" - with a weighting of (100% - sum of likelihoods for probable causes). This approach also facilitates comparisons between physician assessment and computerised probabilistic interpretation of VA material, as recently carried out in South Africa [15].

### Public health interpretation of VA data

In its turn, making epidemiological sense of cause-of-death data is an essential step towards public health understandings of populations. In settings where VA is most practised, which usually lack routine certification, cause-specific mortality findings often represent a key component among relatively scanty overall public health information. Against this background, it is important to consider what the possible adverse effects of different approaches to handling physician opinions on VA data might be.

There are two major issues here. Firstly, the often limited amount of information contained in VA interviews is more likely to leave physician coders in some doubt as to the underlying cause of death, compared with certification in more sophisticated settings, and in turn this is more likely to lead independent coders to different conclusions on the same case. In terms of public health, however, it is clearly unhelpful to completely lose the thinking around such cases by consequently declaring them indeterminate. It is much more important to capture the uncertainties and range of opinions, handle these data epidemiologically, and so enable them to be included in public health thinking. This is an important difference of principle from ICD-10's exclusion of capturing uncertainty at the individual underlying cause code level (even though physicians may admit to being uncertain at the certification stage [16,17]).

Secondly, if physician coders are essentially forced into reaching a single cause of death per case, problems arise in public health interpretations around certain commonly-linked causes of death. The most obvious is the well-documented interaction between tuberculosis and HIV/AIDS. Many single-cause regimes get around the difficulty of this particular example by designating one single cause as "TB/AIDS" but this is methodologically unsatisfactory and can conceal potentially interesting detail [15]. However, within any such set of commonly-linked causes that are handled individually, if a lack of case-level agreement between physicians is considered "indeterminate", then those particular causes involved may be under-represented in aggregate. A common example of this would be diarrhoea or malnutrition as causes of childhood death, where one physician's "diarrhoea" and another's "malnutrition" could result in neither cause contributing to public health conclusions. There is undoubtedly further work to be done in evaluating different approaches to VA interpretation, including WHO's approach based on ICD-10, but also considering what the most practical and meaningful approaches for policymakers may be.

## Conclusion

Avoiding any confusion between majority opinions and scientific truth is a critical issue in handling clinical opinion data. Although these methodological explorations do not necessarily address or solve all the problems of incorporating clinical opinions on cause of death into epidemiological data and public health conclusions, they highlight some of the potential pitfalls. Because physician certification of deaths and physician interpretation of verbal autopsy data are not entirely equivalent procedures, it cannot be assumed that ways of handling differences of opinion, case-level uncertainty and multiple causes should be similar. The possibility of multiple causes per case emerging from VA assessments should be encouraged rather than hidden, even if associated with some degree of uncertainty. In particular, a lack of agreement between physician opinions on a particular case should not be regarded as a reason to effectively exclude such cases as being of "indeterminate" cause. In broader terms, there may well be other areas of epidemiological research involving multiple clinical opinions where similar considerations are relevant.

## List of abbreviations used

ICD-10: International Classification of Diseases 10^th ^revision; TB/AIDS: tuberculosis and/or acquired immune deficiency syndrome; VA: verbal autopsy; WHO: World Health Organization.

## Competing interests

The authors declare that they have no competing interests.

## Authors' contributions

PB was entirely responsible for this paper
